# Congenital Double Lip: A Rare Deformity Treated Surgically

**Published:** 2016-09

**Authors:** Titiksha Aggarwal, Kirti Chawla, Arundeep Kaur Lamba, Farukh Faraz, Shruti Tandon

**Affiliations:** Department of Periodontics, Maulana Azad Institute of Dental Sciences, New Delhi, India

**Keywords:** Double lip, Surgery, Congenital, Acquired, Suturing

## Abstract

Lip is an important aspect of facial features affecting ones personality. A deformity of the lip characterized by excessive tissue sagging below the usual giving it thicker wider appearance is referred to as double lip. It is a rare occurrence with a proposed male predilection. This article is a report of a 20 years old male with this deformity who presented with the complaint of difficult speech and poor aesthetics. There was no other history patient being systemically healthy. It was successively treated with a simple surgical technique without recurrence over a period of 12 months.

## INTRODUCTION

Double lip is an infrequent anomaly involving either or both but mainly the upper lip.^1^ It is characterised by the presence of a fold of excess or redundant hypertrophic tissue on mucosal side of the lip^1^^,^^2^^,^^3^ caused by excessive areolar tissue and non inflammatory labial mucous gland hyperplasia.^1^^,^^4^ It can be congenital or acquired. During foetal period the mucosa of the upper lip is divided into two transverse zones pars glabrosa and pars villosa. Pars glabrosa is the outer smooth zone close to skin. Pars villosa is the inner zone similar to the mucosa of the oral cavity.^5^

Double lip is hypertrophy of the pars villosa thought to arise during the 2^nd^ and 3^rd^ month of gestation. Persistence of exaggerated horizontal sulcus between the pars glabrosa and the pars villosa give rise to the congenital type of double lip.^1^^,^^6^^,^^7^ When the lip is tensed, pars villosa sags below the pars glabrosa giving it characteristic appearance. The congenital type is present since birth,^5^ predominantly associated with upper lip.^6^^,^^8^ It can occur in isolation or as a part of Ascher’s syndrome^4^^,^^9^^,^^10^ which is also associated with euthyroid goitre and blepherochalasis. A few cases involving both lips have also been reported.^3^^,^^11^^-^^13^

Very rarely isolated lower lip involvement has also been seen.^4^^,^^6^^,^^8^ Double lip deformity can be a result of trauma^5^^,^^14^ or oral habits such as sucking lip between diastema^1^ or between ill fitting dentures.^1^^,^^15^ It is sometimes also referred as ‘‘macrocheilia’’ or hamartoma.^16^ Clinically double lip gets prominent when the orbicularis muscle is tensed as during smile or while talking^4^ affecting the overall facial aesthetics*. *It sometimes also interferes with speech and mastication. There is no race or gender predilection^3^^,^^15^^,^^17^^,^^18^ but Palma and Taub, in a recent report in 2009, suggested a male predilection of 7:1.^11^ This report presents a case of surgical management of upper double lip in a 20 year old male patient.

## CASE REPORT

A 20 yr old male patient reported to the department of periodontics with a complaint of difficult speech and poor aesthetics due to enlarged upper lip since childhood.  Clinical examination revealed a thick upper lip even at rest which got accentuated when the patient smiled or showed his teeth, with excessive transverse fold on the mucosal aspect. There was no midline separation of the lip. There was no blepharochalasis (drooping) of the upper eyelids and no thyroid enlargement. There was no other anomaly visible extra orally. 

Intra orally vestibular depth appeared normal with adequate width of attached gingiva in maxillary anterior region. No midline diastema could be appreciated and labial frenal attachment was also normal. Dental occlusion appeared normal except for a slight retroclination of the maxillary anteriors. Patient questionnaire revealed no past history of trauma since childhood. Patient did not get any dental treatment done till date apart from extraction of his right lower back molar two years back. Patient had no associated systemic illness. Thorough scaling and root planning as a part of phase I therapy was instituted to the patient. After a period of one week, lip surgery was planned.

**Fig. 1:**
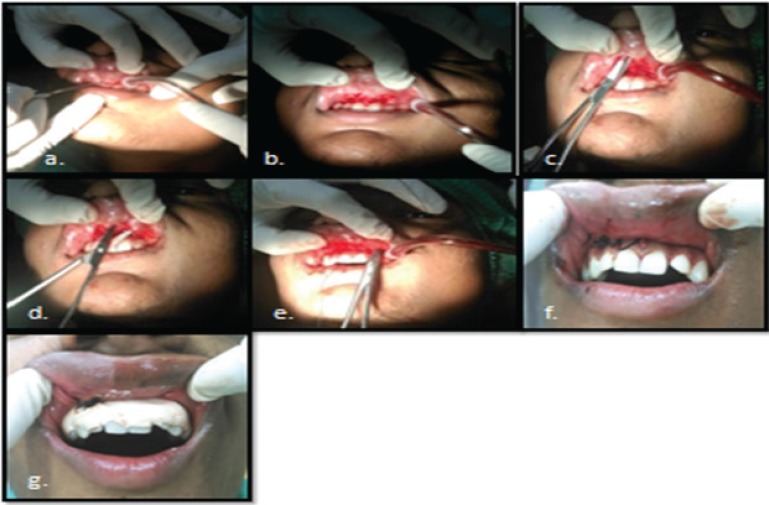
Clinical pictures showing the surgical procedure. a,b: incision, c,d: blind dissection of lip tissue, e,f: suturing, g: periodontal pack placement

**Fig. 2:**
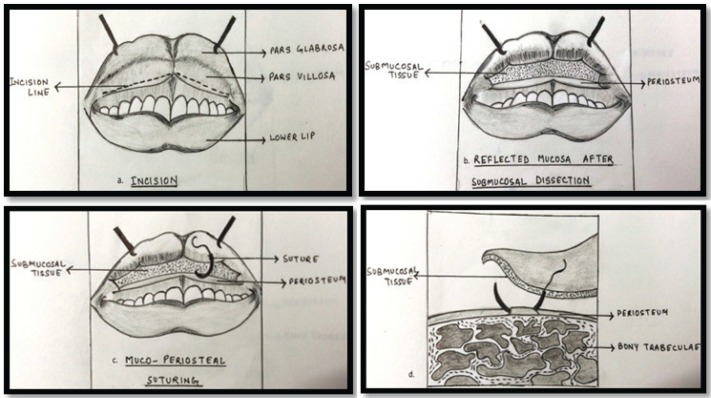
Diagrammatic representation of surgical procedure. a,b: incision and flap reflection, c,d: suturing

Local anaesthesia using 2% lignocaine with adrenaline was instituted. Bilateral infraorbital nerve blocks were given. Vestibular incision using No. 11 Bard Parker blade was made in the vestibule of the upper lip. Supraperiosteal reflection of the tissues was done. Using straight scissor blind dissection of the submucosal tissue of the lip was done. Care was taken to spare the orbicularis muscle fibres of the lip. After the dissection the area was irrigated with normal saline. Using 3-0 black silk suture, the submucosal tissue of pars villosa above the incision line was sutured with the periosteum of the underlying bone. Non eugenol periodontal pack was placed on the surgical site for one week (Figures 1 and 2). 

**Fig. 3:**
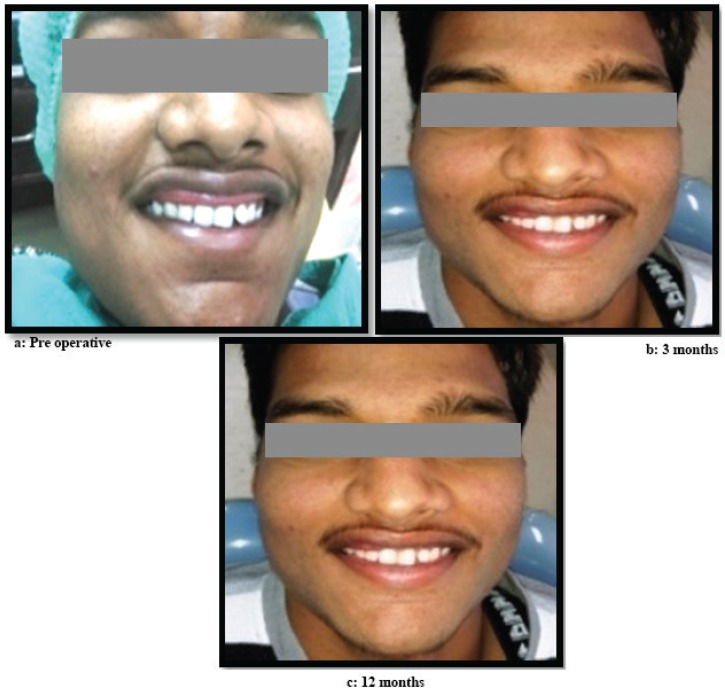
Comparison of pre and post surgical lip profile. a: Pre- operative, b: 3 month post-surgical, c: 12 months post-surgical

Post surgical instructions were given. Capsule amoxicillin with clavulinic acid (625 mg, three times a day), tablet ibuprofen (400 mg, two times a day for three days) and 0.2% chlorhexidine mouthwash for 10 days were prescribed. Sutures with periodontal pack were removed on 7^th^ day of surgery. Thorough irrigation of the wound site with 0.1% povidone iodine was done. Oral hygiene instructions were reinforced to the patient. Recall follow ups were done at the end of 1 and 3 months and 1 year (Figure 3).

## DISCUSSION

Congenital double lip is non-inflammatory enlargement of the lip either due to glandular tissue hyperplasia or due to persistence of the horizontal sulcus between the developing parts of lip namely pars glabrosa and pars villosa during second to 3^rd^ week of gestation. Single uniform enlargement of the lip tissue^5^^,^^19^ and those with central constriction due to upper labial frenum have also been reported.^4^^,^^6^^,^^8^^,^^9^^,^^19^ Such enlargements are usually bilaterally symmetrical but a few unilateral enlargement cases have also been reported.

Clinical features of congenital double lip usually become apparent after the eruption of the permanent teeth.^1^^,^^8^ It is generally reported that the upper double lip is not evident at rest but when the lip is tensed as during smiling, laughing or attempting to show teeth.^5^^,^^19^ Reason being the contractions of the muscle orbicularis oris exaggerates the horizontal sulcus, retracts the lip and places the mucosa over the maxillary teeth giving a double lip appearance.^1^ Although, few cases of double lip appearance have been reported who show the deformity even at rest,^20^ current report has highlighted the features of upper lip with uniform sagging of pars villosa which became prominent when lip was tensed.

Double lip has been shown to be associated with some syndromes. Laffer in 1909^4^ described double lip associated with blepherochalasis. Ascher an ophthalmologist in 1920 described a triad of double upper lip, blepherochalasis and non-toxic thyroid enlargement. It is transmitted as autosomal dominant disorder exact aetiology is not known.^5^ Subject in the current report did not show any of the features of blepharochalasis or thyroidism so syndromic aetiology was ruled out.

Most cases of this condition are associated with aesthetic concern; very few also show functional interference. Management is aimed at both the functional and aesthetic improvement. Surgery is the only way out to treat the condition till date. Reduction in the height of the lip is the aim of the surgery with the key area of focus being sutures. Vicryl or polyglactin is the preferred suture for subcutaneous tissues, but due its inability to withstand enough mechanical load (produced by orbicularis oris and hyperplastic tissue), 3-0 black silk sutures were used in the present study. The submucosal tissue of pars villosa after blind dissection was sutured with the underlying periosteum over the labial cortical plate near the apices of the maxillary incisors. 

No excess tissue was extracted from the surgical site. Carefully all the orbicularis oris muscle fibres were spared while suturing. Muscle fibres can interfere with healing by mobilising the site leading to inappropriate outcomes like recurrence. Another important aspect of the surgery is the choice of anaesthesia. Most plastic surgery procedures are done under general anaesthesia, but it has its own necessities and limitations. Among the local anaesthetic techniques choice between local infiltration and nerve block is crucial to this surgery. Preferred anaesthesia is infraorbital nerve block. Local infiltration cause tissue distortion and can produce a false exaggerated double lip appearance which can cause the surgeon to either under or overestimate the extent of deformity.

The present technique could successfully manage the deformity with no intra or postoperative complications. The above was a simple technique with good aesthetic outcome. No recurrence was observed within a period of 12 months. Although a longer follow up may be required to check for any recurrence.
